# Oxidative Stress and Inflammation in Hypoxemic Respiratory Diseases and Their Comorbidities: Molecular Insights and Diagnostic Advances in Chronic Obstructive Pulmonary Disease and Sleep Apnea

**DOI:** 10.3390/antiox14070839

**Published:** 2025-07-08

**Authors:** Jorge Rodríguez-Pérez, Rosa Andreu-Martínez, Roberto Daza, Lucía Fernández-Arroyo, Ana Hernández-García, Elena Díaz-García, Carolina Cubillos-Zapata, Alicia Lozano-Diez, Aythami Morales, Daniel Ramos, Julián Aragonés, Ángel Cogolludo, Luis del Peso, Francisco García-Río, María J. Calzada

**Affiliations:** 1 Department of Medicine, School of Medicine, Universidad Autónoma de Madrid, 28049 Madrid, Spain; jorge.rodriguezperez@estudiante.uam.es (J.R.-P.); rosa.andreu@estudiante.uam.es (R.A.-M.); lucia.fdez11@gmail.com (L.F.-A.); julian.aragones@uam.es (J.A.); 2 Instituto Investigación Sanitaria-Princesa IIS-IP, 28006 Madrid, Spain; 3 Biometrics and Data Pattern Analytics Lab, Escuela Politécnica Superior, Universidad Autónoma de Madrid, 28049 Madrid, Spain; roberto.daza@uam.es (R.D.); aythami.morales@uam.es (A.M.); 4 Department of Pharmacology and Toxicology, School of Medicine, Universidad Complutense de Madrid, 28040 Madrid, Spain; anaher10@ucm.es (A.H.-G.); acogolludo@med.ucm.es (Á.C.); 5 Centro de Investigación Biomédica en Red de Enfermedades Respiratorias (CIBERES), Instituto de Salud Carlos III, 28029 Madrid, Spain; elena.diaz.garcia.1994@gmail.com (E.D.-G.); cubillos.zapata@gmail.com (C.C.-Z.); francisco.garcia@uam.es (F.G.-R.); 6 IdiPAZ, Instituto de Investigación Sanitaria del Hospital Universitario La Paz, 28029 Madrid, Spain; luis.peso@uam.es; 7 AUDIAS—Audio, Data Intelligence and Speech, Escuela Politécnica Superior, Universidad Autónoma de Madrid, 28049 Madrid, Spain; alicia.lozano@uam.es (A.L.-D.); daniel.ramos@uam.es (D.R.); 8 Centro de Investigación Biomédica en Red de Enfermedades Cardiovasculares (CIBERCV), Instituto de Salud Carlos III, 28029 Madrid, Spain; 9 Instituto de Investigaciones Biomédicas Sols-Morreale (IIBM), Consejo Superior de Investigaciones Científicas, Universidad Autónoma de Madrid, Arturo Duperier, 4, 28029 Madrid, Spain

**Keywords:** chronic obstructive respiratory disease, obstructive sleep apnea, hypoxia, HIF, oxidative stress, inflammation, comorbidities, cancer, pulmonary hypertension, artificial intelligence

## Abstract

In chronic respiratory diseases (CRDs), oxidative stress and inflammation are closely linked, driving disease onset, progression, and comorbidities. Oxidative stress activates inflammatory pathways, while chronic inflammation promotes further reactive oxygen species (ROS) production, creating a vicious cycle leading to airway remodeling, reduced lung function, and exacerbations. This review highlights the central roles of inflammation and oxidative stress in chronic obstructive pulmonary disease (COPD) and obstructive sleep apnea (OSA). In COPD, chronic hypoxemia associates with emphysema, appearing with disease progression. In OSA, beyond systemic consequences, pulmonary inflammation and oxidative stress contribute to lung injury as well. Although COPD and OSA are distinct conditions, some patients present with “overlap syndrome”, a term used in this review to describe the coexistence of both. This combination poses unique diagnostic and therapeutic challenges. We also examine the role of hypoxia and its transcriptional effects via hypoxia-inducible factors (HIFs) in promoting oxidative stress and inflammation. Finally, we explore how artificial intelligence (AI) offers promising tools to improve diagnosis, monitoring, and management of CRDs and may help elucidate mechanistic links between hypoxia, inflammation, and oxidative stress, contributing to more personalized therapeutic strategies.

## 1. Introduction

Globally, chronic respiratory diseases (CRDs) are estimated to cause one death every 10 s (equivalent to 3 million annually) and are projected to become the third leading cause of mortality by 2030, according to the World Health Organization (WHO). Data from the Global Burden of Diseases, Injuries, and Risk Factors Study [1] indicates that total deaths and the prevalence of CRDs have increased by 28.5% and 39.8%, respectively [1]. Chronic obstructive pulmonary disease (COPD) and obstructive sleep apnea (OSA) are highly prevalent chronic respiratory conditions associated with significant morbidity. The global prevalence of COPD among adults is estimated at approximately 12% [2,3], whereas the prevalence of OSA may reach up to 38% [4]. Both conditions are characterized by reduced arterial oxygen tension (hypoxemia), increased production of ROS, and an exacerbated inflammatory response, all of which shape the clinical manifestations observed in these patients. Notably, the clinical course of these individuals is highly variable and largely influenced by the development of comorbidities, including cardiovascular, metabolic, and neoplastic diseases, that are often the primary cause of mortality. Despite this, the underlying mechanisms that govern the onset and progression of these comorbidities remain poorly defined, and robust biomarkers to predict comorbidome risk have yet to be identified.

COPD is defined by the presence of respiratory symptoms and persistent airflow limitation resulting from structural abnormalities in the airways and/or alveoli [5]. Although traditionally associated with older adults, COPD can also arise from early-life developmental alterations [6,7]. While smoking remains the primary etiological factor and is strongly associated with adverse clinical outcomes and increased mortality in genetically susceptible individuals worldwide, only approximately 15% of smokers develop an exaggerated local inflammatory response to ROS in cigarette smoke. This leads to irreversible pulmonary damage [8,9] and the emergence of a systemic proinflammatory state [10]. This pathological condition contributes to the progression and prognosis of COPD, promoting the development of comorbidities linked to systemic dysfunction, reduced quality of life, increased hospitalizations, suboptimal therapeutic responses, and elevated mortality rates [11,12].

Frequent comorbidities observed in COPD patients include cardiovascular diseases, metabolic disorders, pulmonary fibrosis, and lung cancer [13,14,15]. It is estimated that 78.6% of individuals with COPD present with at least one clinically relevant comorbidity, 68.8% with two or more, and nearly 47.9% with three or more. Although patients with advanced airflow limitation often succumb to respiratory failure, the majority of deaths among COPD patients are due to non-respiratory causes, chiefly cardiovascular disease and cancer [14,16]. Despite these clinical correlations, the pathophysiological mechanisms underlying the association between COPD and its comorbidities remain poorly understood. Beyond aging, sedentary lifestyle, and tobacco use, both hypoxia and systemic inflammation, hallmarks of COPD, are believed to significantly contribute to the high burden of comorbid conditions in this population [3,17].

OSA is another prevalent respiratory disorder characterized by repeated collapse of the upper airways during sleep, leading to intermittent hypoxia (IH), periodic drops in oxygen saturation, and sleep fragmentation (micro-awakenings). These disturbances result in non-restorative sleep, causing fatigue and daytime sleepiness, and contribute to the development of respiratory, neuropsychiatric, metabolic, cardiovascular, and oncological disorders [18]. OSA is also associated with a high mortality rate, primarily linked to daytime sleepiness and the onset of comorbidities, particularly cardiovascular and metabolic conditions [19,20,21,22]. Severe OSA has been specifically associated with an increased risk of dyslipidemia, non-alcoholic fatty liver disease, and, most notably, diabetes [23]. Additionally, it is also associated with a higher risk of arrhythmias, hypertension, atherosclerosis, ischemic heart disease, and stroke [24,25]. Although the high prevalence of hypertension in OSA patients, exceeding 50% in some cohorts, has been proposed as the main driver of its cardiovascular complications [26], other risk factors, particularly those promoting atherosclerosis, also play a critical role [27,28]. Among these, dyslipidemia and inflammation, both highly prevalent in OSA, stand out as key proatherosclerotic contributors [29,30].

Moreover, several epidemiological studies, patient cohort analyses, and animal models have shown that OSA is associated with an increased incidence, aggressiveness, and mortality of various types of cancer [31,32]. IH has been proposed as a potential mechanism, promoting tumor vascularization, facilitating tumor growth, and modulating immune responses by disrupting immune surveillance, thus allowing the emergence of more aggressive tumors [33,34,35]. In addition, the repetitive cycles of hypoxia and reoxygenation alter redox homeostasis in OSA, promoting ROS production and reducing endogenous antioxidant defenses. This oxidative imbalance correlates with disease severity [36].

Overlap syndrome, as commonly known to refer to the condition of both COPD and OSA, is relatively frequent and is characterized by nocturnal hypoxemia. This condition leads to worse clinical outcomes than either disease on its own [37]. Recent studies have demonstrated that patients with overlap syndrome exhibit elevated levels of systemic inflammatory biomarkers compared to individuals with isolated COPD or OSA, potentially contributing to the increased cardiovascular risk observed in this subgroup [38]. However, inflammation alone may not fully account for the heightened cardiovascular risk, highlighting the need for further investigation into alternative pathogenic mechanisms.

In the context of CRDs, persistent hypoxic conditions trigger a key adaptive response mediated by transcriptional programs governed primarily by hypoxia-inducible factors (HIFs). HIF-1α and HIF-2α are central mediators of cellular adaptation to low oxygen levels, regulating a wide array of genes involved in essential adaptive processes [39,40]. However, in the context of CRDs, this regulation is complex and significantly contributes to disease pathogenesis and progression. While initially protective, chronic activation of HIF pathways can become maladaptive, initiating a cascade of detrimental effects. In this review, we highlight several HIF-mediated pathophysiological responses, with a particular focus on pulmonary vascular adaptation to hypoxia.

Despite advances in understanding the pathophysiological mechanisms underpinning COPD and OSA, substantial gaps remain in early diagnosis, prognosis, and personalized treatment approaches. The complexity and heterogeneity of CRDs, particularly the interplay between hypoxia, oxidative stress, inflammation, and comorbidities, pose significant challenges for conventional clinical methods. In this context, artificial intelligence (AI) has emerged as a promising solution to overcome these limitations. By integrating and analyzing large-scale, multimodal clinical and molecular data, AI tools offer the potential to improve diagnostic accuracy, stratify patient risk, and unveil novel insights into disease mechanisms. These capabilities make AI particularly valuable for refining our understanding and improving diagnosis and prognosis in diseases such as COPD [41] and OSA [42], as well as the underlying molecular pathways and its use in personalized medicine. Advancing AI systems in respiratory medicine require multidisciplinary teams that bring together clinicians, data scientists, engineers, and ethicists to ensure that models are both technically robust and clinically relevant.

## 2. Transcriptional Response to Sustained and Intermittent Hypoxia in CRDs

As we have mentioned, despite their diverse etiology and clinical manifestations, both OSA and COPD share a common feature: the development of hypoxemia. While in COPD these hypoxic conditions are primarily caused by alveolar damage, known as emphysema, in the case of OSA intermittent hypoxia due to repeated upper airway obstruction during sleep results in temporary drops in oxygen saturation. Although the degree and duration of oxygen desaturation vary widely depending on disease severity and adherence to treatment, some degree of tissue hypoxia is expected in the majority of cases [43,44]. Moreover, tissue hypoxia is increasingly recognized as a potential major contributor to the pathogenesis of chronic respiratory diseases (CRDs) [43,45].

To maintain oxygen homeostasis, the body employs various mechanisms, broadly categorized into immediate physiological adjustments [46] and longer-term transcriptional responses [39]. Acute responses are triggered within seconds of oxygen deprivation, such as the activation of chemoreceptors like the carotid body to modulate respiration, and vascular changes that enhance oxygen delivery. However, these mechanisms are insufficient for adaptation to prolonged hypoxia. Instead, transcriptional responses become central over hours or days, adjusting metabolism and oxygen supply and playing a critical role in adapting to CRDs. Additionally, hypoxia-induced transcriptional reprogramming may help precondition tissues to better tolerate future hypoxic episodes [47,48], providing an essential adaptive mechanism in chronic respiratory conditions where tissues are repeatedly subjected to low oxygen levels.

### 2.1. HIF Signaling and Gene Expression Dynamics in Sustained and Intermittent Hypoxia

A universal property of nucleated cells is their ability to autonomously sense oxygen levels and regulate members of the HIF family of transcription factors, which orchestrate a broad gene expression response to hypoxia. HIFs, consisting of an oxygen-regulated alpha subunit and a constitutively expressed beta subunit (aryl hydrocarbon receptor nuclear translocator, ARNT), drive a metabolic shift from oxidative phosphorylation to glycolysis, reducing both oxygen consumption and the generation of ROS. They also promote the transcription of genes involved in angiogenesis and erythropoiesis, facilitating oxygen delivery to hypoxic tissues [39]. While these adaptations enhance survival, as mentioned above chronic HIF activation is implicated in pathological processes including tissue remodeling, inflammation, and abnormal vascularization, hallmarks of chronic respiratory and cardiovascular diseases [40].

The oxygen-dependent regulation of HIF and its role in hypoxia adaptation have been extensively characterized, a contribution recognized by the 2019 Nobel Prize in Physiology or Medicine. The HIF pathway depends on a set of oxygen-sensitive enzymes that act as cellular oxygen sensors [49]. Under normoxic conditions, EGLN family prolyl hydroxylases hydroxylate specific proline residues on HIF-α, marking it for recognition by a ubiquitin ligase complex containing the von Hippel–Lindau (VHL) tumor suppressor protein and targeting it for proteasomal degradation [49]. These EGLN enzymes require iron (Fe^2+^), 2-oxoglutarate, and molecular oxygen for their activity, making them highly sensitive to oxygen levels. Even mild hypoxia inhibits EGLN activity, leading to HIF-α stabilization and activation of its downstream transcriptional targets. HIF transcriptional activity is also regulated by another oxygen-dependent enzyme factor inhibiting HIF (FIH), which hydroxylates an asparagine residue on HIF-α [49]. The central role of HIFs in hypoxia is further reinforced by early studies showing that most hypoxia-induced genes are dependent on HIF activity [50].

Nevertheless, while HIFs are central players, other transcription factors also contribute to the hypoxic gene expression program by integrating diverse cellular signals. NF-κB is a key example, especially relevant for inflammatory responses to hypoxia. Its activation is closely linked to the inhibition of EGLN enzymes, paralleling HIF regulation [51]. Importantly, a recent report suggests a large part of the hypoxia-regulated genes depend, at least in part, on NF-κB [52]. This interplay between NF-κB and HIF is particularly significant in CRDs, where inflammation and hypoxia coexist, with NF-κB acting as a key transcription factor in inflammatory responses and HIF playing a central role in the cellular response to hypoxia. FOXO3 is another transcription factor regulated under hypoxia through prolyl hydroxylation [53]. This factor modulates responses such as oxidative stress resistance and metabolic adaptation [54]. Notch signaling also intersects with hypoxic pathways via direct interactions between HIF-1α and the Notch intracellular domain (NICD), resulting in the hypoxic induction of Notch target genes [55]. Direct hydroxylation of NICD in an oxygen-dependent manner has also been reported. AP-1, in contrast, becomes more prominent following prolonged hypoxic exposure [56], suggesting a role in later stages of adaptation rather than in the immediate response [57,58]. Similarly, the Hippo pathway, which regulates transcriptional enhanced associated domain (TEAD) transcription factors, is modulated by oxygen levels, potentially through the hypoxia-inducible ubiquitin ligase Siah2 [59]. This interaction may contribute to the development of pulmonary hypertension [60,61] and other cardiovascular diseases [62], which are common comorbidities associated with CRDs. Other transcription factors, such as TP53 and Nrf2, may respond to hypoxia in more context-dependent ways. TP53 is generally induced in severe hypoxia or anoxia, which may explain conflicting findings regarding its regulation by oxygen [63]. Nrf2, a master regulator of oxidative stress, is also likely to be indirectly involved in hypoxia adaptation [64], particularly during reoxygenation and in conditions that promote ROS production [65]. Given the critical role of ROS in the progression of CRDs, the involvement of Nrf2 in hypoxia-related responses, albeit indirect, may be especially important in this context. Thus, while HIFs are central, a diverse network of transcription factors contributes to the hypoxic response, integrating cues from inflammation, metabolism, and cellular stress.

Beyond transcription factor regulation, oxygen availability also influences gene expression via epigenetic mechanisms, adding another layer of dynamic control during hypoxic adaptation. Epigenetic modifications are crucial for modulating hypoxia-induced gene expression by altering chromatin accessibility. Seminal studies have demonstrated that hypoxia inhibits histone demethylases such as KDM5A and KDM6A, leading to increased levels of histone marks like H3K4me3 and H3K27me, respectively, modifications that contribute to hypoxic responses independently of HIF [66,67]. These and other findings indicate that DNA and histone methylation states shift dynamically in response to oxygen levels. Many demethylases are 2-oxoglutarate-dependent dioxygenases that require oxygen as a cosubstrate, positioning them as oxygen sensors analogous to HIF prolyl hydroxylases [68,69]. Members of the KDM4, KDM5, and KDM6 families, which have a high affinity for oxygen, are particularly susceptible to inhibition under physiological hypoxia. As a result, hypoxia can profoundly alter chromatin landscapes, modulating gene expression in ways that extend beyond the HIF pathway [68,69]. These insights highlight the importance of oxygen-dependent epigenetic regulation in shaping hypoxic responses and potentially influencing long-term disease progression.

The convergence of these transcriptional and epigenetic regulators underscores the complexity of the cellular response to hypoxia and the importance of considering multiple regulatory pathways. However, caution is warranted when extrapolating findings to CRDs. A significant limitation is that much of the current knowledge comes from in vitro cellular models, often derived from tumor cell lines that are not representative of lung tissue. This raises uncertainty regarding the contribution of these transcriptional mechanisms to hypoxia adaptation in pulmonary cell types in vivo. Moreover, most studies focus on sustained hypoxia, even though oxygen levels fluctuate in CRDs. OSA represents an extreme case, with severe patients experiencing more than 30 apnea episodes per hour, and some exceeding 80 [70]. These cycles of hypoxia and reoxygenation create a physiological context distinct from continuous hypoxia. Whether IH elicits the same transcriptional program as sustained hypoxia remains unclear, especially in cases involving high-frequency, short-duration hypoxic episodes.

### 2.2. Role of HIFs in Vascular Dysfunction in COPD and OSA

Hypoxic conditions in the vascular endothelium trigger the proliferation of smooth muscle cells, increasing their coverage around pulmonary vessels. This vascular remodeling raises pulmonary arterial pressure, potentially leading to right ventricular hypertrophy and, ultimately, heart failure. Previous studies have demonstrated that hypoxia-induced pulmonary vascular remodeling is mediated by HIFs. Specifically, HIF-induced vascular remodeling involves the emergence of pulmonary artery smooth muscle cells (PASMCs), which results in the muscularization of alveolar wall vessels resulting in increased pulmonary arterial pressure. This vascular remodeling leads to the thickening of the tunica media and adventitia of pulmonary arteries.

In this context, mice with heterozygous germline deletion of HIF-1α or HIF-2α exhibit attenuated development of pulmonary hypertension upon chronic hypoxic exposure [71,72,73]. Further investigations have confirmed the pivotal role of endothelial HIF-2α in hypoxia-driven pulmonary hypertension in which endothelial-cell-specific deletion of HIF-2α protects against the development of hypoxia-induced pulmonary hypertension [49,74,75,76,77,78]. Conversely, sustained activation of HIF-2α in pulmonary artery endothelial cells in mice leads to pulmonary hypertension and right ventricular hypertrophy [74,77,79].

**Figure 1:**
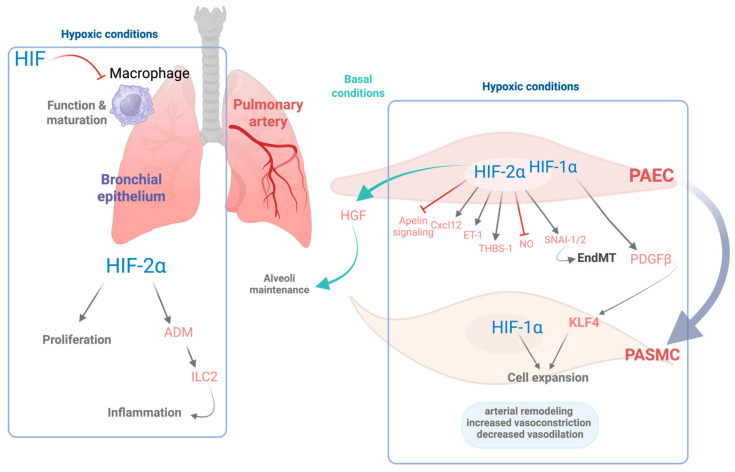
Role of HIFs in pulmonary cellular responses and vascular remodeling. This schematic illustrates the cellular and molecular effects of hypoxia mediated by HIF-1α and HIF-2α isoforms as well as their role in basal conditions in various pulmonary cell types in baseline and hypoxic conditions. Under hypoxic conditions, HIF signaling is activated, orchestrating the transcription of numerous genes that contribute to pulmonary arterial remodeling and favor vessel contraction. In pulmonary artery endothelial cells (PAECs), HIF-2α regulates both pro- and antiremodeling pathways, including the induction of CXC motif chemokine ligand 12 (CXCL12), endothelin-1 (ET-1), thrombospondin-1 (THBS-1), and the zinc-finger transcription factors SNAI-1/2, which contributes to endothelial-to-mesenchymal transition (EndMT). Conversely, HIF-2α concurrently inhibits apelin and nitric oxide (NO) signaling. In turn, endothelial HIF-1α stimulates the release of platelet-derived growth factor beta (PDGFβ) leading to the activation of the pluripotency factor Krüppel-like factor 4 (KLF4) in both PAECs and PASMCs, which together with HIF-1α activation promotes expansion of PASMCs and vascular remodeling. Collectively, these responses lead to increased vasoconstriction, impaired vasodilation, and structural changes in the pulmonary vasculature. Under basal oxygen conditions, endothelial HIF-2α plays homeostatic roles, supporting alveolar maintenance via hepatocyte growth factor (HGF). Moreover, in the bronchial epithelium, HIF-2α induces proliferation of the bronchial epithelial cells as well as increased expression of adrenomedullin (ADM), which activates the group 2 innate lymphoid cells (ILC2s), contributing to Th2 airway inflammation. In the alveolar macrophages, HIF signaling counteracts macrophage maturation and function. Overall, this figure highlights the complementary and context-dependent roles of HIF-1α and HIF-2α in pulmonary adaptation to hypoxia, with critical implications for inflammation and vascular remodeling. Figure created with Biorender.com.

In line with these findings, both human studies and mouse models carrying gain-of-function mutations in HIF-2α have demonstrated severe pulmonary hypertension phenotypes [80,81]. Additionally, HIF-2α regulates the expression of key endothelial genes that enhance vasoconstriction and impair vasodilation, including endothelin-1, arginase activity, as well as suppression of apelin signaling. It also upregulates molecules such as CXC motif chemokine ligand (CXCL12), which promotes the proliferation and migration of pulmonary vascular smooth muscle cells [77,78,82]. HIF-2α has been shown to upregulate the expression of thrombospondin-1 (TSP-1) in hypoxic lungs, contributing to vascular remodeling and vasoconstriction [83]. In addition, HIF-2α has the potential to induce the expression of the transcription factors SNAI-1/2, key regulators of endothelial-to-mesenchymal transition (EndMT), which in turn contributes to pulmonary vascular remodeling [84]. In parallel, HIF-1α activity in both endothelial and smooth muscle cells has been shown to be implicated in the pathogenesis of hypoxia-induced pulmonary hypertension. Specifically, HIF-1α drives the expression of platelet-derived growth factor B (PDGF-B) in endothelial cells, which in turn promotes the expansion of PASMC via activation of the pluripotency factor Krüppel-like factor 4 (KLF4) [73,85] (see Figure 1). Apart from the role of HIF-2α overactivation in pulmonary hypertension, decreased HIF-2α activity in lung endothelial cells also plays an important role in other pathological conditions, including COPD. Notably, these patients exhibit decreased expression of HIF-2α in the lungs [86]. Consistent with these findings, an independent study further demonstrated that lung tissue from COPD patients contains a reduced number of CD31^+^ endothelial cells, along with lower HIF-2α expression per CD31^+^ cell, an effect that is particularly pronounced in advanced stages of the disease [87]. Similarly, diminished endothelial HIF-2α expression has been observed in murine models of COPD following chronic exposure to cigarette smoke [86,87].

In agreement with these results, genetic inactivation of HIF-2α specifically in pulmonary endothelial cells results in an emphysematous phenotype characterized by enlarged alveolar spaces and increased apoptosis of alveolar structural cells [87]. These findings suggest that reduced HIF-2α expression in pulmonary endothelial cells may act as a pathogenic driver in the progression of COPD. Moreover, its deficiency in lung endothelial cells leads to a concomitant reduction in both endothelial cells and pericytes. This is associated with diminished expression of hepatocyte growth factor, a key mediator involved in the preservation of the alveolar architecture by sustaining vascular survival [86]. Importantly, this emphysematous phenotype is not observed following endothelial-specific deletion of HIF-1α, underscoring the pivotal role of HIF-2α in preserving lung vascular homeostasis [87]. Collectively, these findings support the idea that endothelial HIF-2α plays a critical protective role in maintaining alveolar architecture and limiting pathological inflammation in the lung. Nevertheless, excessive HIF-2α activation in endothelial cells, such as that observed in pulmonary hypertension, can be detrimental, which highlights the importance of well-balanced HIF-2α levels in the endothelium [86]. A detailed scheme of HIF signaling within the lung is included in Figure 1.

In addition to the role of the HIF pathway in pulmonary hypertension, HIF isoforms have been also involved in systemic hypertension in OSA [88]. Indeed, IH-dependent HIF-1α activity has been proposed to hyperactivate the central and peripheral nervous system leading to hypertension. At a molecular level HIF-1α induces the prooxidant protein NADPH oxidase (NOX)-2, which provokes oxidative stress and subsequent inhibition of heme-oxygenase 2 and generation of hydrogen sulfide [88,89]. In contrast, HIF-2α counteracts this pathological action of HIF-1α by inducing the antioxidant enzyme SOD2, which can counteract HIF-1α-dependent oxidative stress. In this line, IH evoked a degradation of HIF-2α therefore facilitating oxidative-stress-dependent hypertension [90]. In addition to the central and peripheral nervous system, IH induces HIF-1α in heart and aortic tissue, which leads to oxidative stress, vascular smooth muscle cell migration, vascular remodeling, and systemic hypertension [91]. This HIF-1α-dependent vascular remodeling has been associated with the activation of the Hippo-YAP pathway [91].

### 2.3. Role of HIF Pathway in Airway Inflammation

HIF signaling is also activated in other pulmonary cell types, including epithelial Club cells, where HIF-2α promotes epithelial cell proliferation [92]. Notably, the progression of COPD is associated with a chronic inflammatory state in the airways [93], a process in which HIF activity may play a significant role. Supporting this notion, activation of HIF-2α in Club cells triggers a type 2 helper T cell (Th2)-skewed inflammatory response in the lung, characterized by the activation of group 2 innate lymphoid cells (ILC2s) [94]. At the molecular level, this effect is mediated through HIF2α-induced expression of adrenomedullin (ADM), a gene which contains a hypoxia response element (HRE) in its proximal promoter [94]. ADM enhances ILC2 activation by increasing the expression of surface markers such as Sca-1 and KLRG1 and by amplifying their capacity to secrete IL-5 [94] (see Figure 1).

These findings establish a novel link between HIF-2α activity, ILC2 biology, and Th2-driven airway inflammation. Additionally, recent clinical evidence demonstrates that patients with severe COPD present elevated numbers of ILC2s in sputum samples [95]. In parallel, circulating ADM levels have been identified as a potential biomarker for COPD exacerbation [96], while proadrenomedullin (proADM) has been proposed as a predictor of survival in COPD patients [97]. Collectively, these data suggest that aberrant HIF-2α activity in the bronchial epithelium may exacerbate COPD by promoting airway inflammation through the ADM–ILC2 axis. Conversely, evidence suggests that IH preferentially activates NF-κB over HIFs, particularly in OSA models, where inflammation rather than canonical HIF-driven adaptation appears to dominate [98]. Supporting this, IH has been associated with NF-κB-mediated inflammatory gene expression in adipose tissue, a key contributor to the cardiovascular complications of OSA [99]. These observations highlight the need for further studies to systematically compare the transcriptional effects of sustained versus IH, particularly in CRDs, where oxygenation patterns have critical implications for disease progression and treatment strategies.

In addition to epithelial cells, HIF transcription factors also modulate immune responses in alveolar macrophages, which are essential for maintaining immune homeostasis within the alveolar space. HIF-1α expression in myeloid cells has been shown to be critical for sustaining inflammatory responses [100], with HIF-1α generally associated with M1 macrophage polarization, whereas HIF-2α has been linked to M2 polarization [101]. However, this paradigm appears more complex in the context of alveolar macrophages. Constitutive activation of HIF-1α signaling, through deletion of tumor suppressor gene VHL, leads to impaired Th2 responses, evidenced by reduced eosinophil recruitment and lower IL-5 and IL-13 production [102]. Furthermore, VHL inactivation in alveolar macrophages induces an immature phenotype and impairs their capacity for surfactant clearance [103] (see Figure 1).

These findings indicate that, contrary to its role in promoting proinflammatory M1 phenotypes in other contexts, HIF-1α may act by suppressing specific immune functions of alveolar macrophages. Importantly, alveolar macrophage function is known to be compromised in COPD [104]; however, the potential contribution of HIF signaling to this dysfunction remains unexplored and requires further investigation.

## 3. Role of Oxidative Stress and Inflammation in CRDs

As mentioned above, oxidative stress is a key mechanism in CRD development, making the identification of oxidative-stress-related biomarkers beneficial for improving its diagnosis and treatment. Repeated cycles of hypoxia and reoxygenation, along with exposure to indoor and outdoor air pollutants and other toxic chemicals, are key factors that stimulate the endogenous production of ROS in respiratory diseases such as OSA and COPD [8,105,106]. At low concentrations, ROS act as secondary messengers, playing a crucial role in redox signaling and regulating a variety of essential physiological processes, including bacterial killing during phagocytosis, vasodilation, tissue repair, and regeneration [107,108]. However, when present at elevated levels, ROS can cause irreversible damage to cellular biomolecules, such as lipids, carbohydrates, and nucleic acids, impairing their function, altering cellular metabolism, and ultimately leading to cell and tissue injury [105]. Importantly, oxidative stress and inflammation are not isolated processes but mutually reinforcing: oxidative stress activates inflammatory responses, which in turn further enhance ROS production, creating a self-sustaining pathogenic loop. This dynamic interplay contributes to the onset and progression of chronic respiratory diseases and has been increasingly recognized as a non-linear, bidirectional process [109,110].

Recent findings from multivariate logistic regression models have revealed a negative correlation between oxidative balance scores and the likelihood of COPD prevalence [111]. This imbalance is partly attributable to free radicals in cigarette smoke, which enhance endogenous ROS production and trigger inflammatory responses at the cellular level. Consequently, the respiratory systems of smokers exhibit age-related characteristics, partially due to increased oxidative stress, accumulation of damaged proteins, and elevated inflammation. On this matter, expression of the ROS-generating enzyme NOX4 is enhanced in airway smooth muscle from patients with COPD, which is also correlated with disease severity and lung function decline [112]. Indeed, in end-stage COPD, not only NOX4 but also other isoforms such as NOX1, NOX2, and NOX5 remain active. Among them, NOX1 and NOX4 are notably involved in oxidative stress and inflammation following acute exposure to cigarette smoke [113]. Thus, NOX enzymes are considered potential therapeutic targets for COPD. Supporting this, apocynin, a selective NOX inhibitor, partially improved cigarette-smoke-induced lung neutrophilia and reversed systemic inflammation and oxidative stress [114] in mice, although clinical trials have not been reported yet in COPD. Conversely, others demonstrate protective roles for certain NOX/DUOX family members in COPD models [115,116,117]. These findings suggest that non-specific antioxidant therapies may be ineffective or even counterproductive.

COPD patients exhibit a distinctive inflammatory profile characterized by elevated levels of macrophages, neutrophils, and both T and B lymphocytes in airway secretions [118,119]. This inflammatory response involves both the innate and adaptive immune systems, interconnected through dendritic cell activation. Compared to smokers without airway obstruction, COPD patients show a heightened inflammatory response that persists even after smoking cessation [120]. Although smoking is the major environmental risk factor, only a subset of smokers develop COPD, likely due to genetic predispositions, epigenetic modifications, and oxidative stress, all of which may amplify inflammation [119]. Persistent oxidative stress and inflammation accelerate epithelial cell senescence, weaken barrier integrity, and drive chronic airway inflammation and tissue remodeling [121]. During acute exacerbations, systemic inflammation often worsens [122,123], and population studies link elevated systemic inflammatory markers to higher risks of diabetes, cardiovascular disease, lung cancer, and mortality [120,124].

OSA is another chronic respiratory condition wherein oxidative stress and inflammation play significant roles. The cyclical pattern of oxygen deprivation and reoxygenation intrinsic to OSA leads to excessive ROS production, overwhelming cellular antioxidant defenses and triggering oxidative stress. Such stress contributes to OSA-related complications, including cardiovascular disorders [36,125]. Despite evidence of elevated ROS in OSA, no consensus exists on reliable oxidative stress biomarkers, highlighting the need for further research. Unlike continuous hypoxia, OSA-associated IH includes a posthypoxic reoxygenation phase, marked by frequent, severe oxygen desaturation and fluctuations [126]. This pattern amplifies ROS production, enhancing oxidative stress and inflammation. Oxidative stress also activates NF-κB, promoting the release of proinflammatory cytokines such as IL-1α, IL-1β, IL-6, and TNF-α [127,128]. Recent studies demonstrate that IH in OSA patients induces cardiomyocyte apoptosis, through the calcium-permeable cation channel TRPC5, contributing to increased ROS production, disrupting mitochondrial function, and disturbing calcium balance. These findings shed light on the mechanisms of TRPC5-mediated cardiac damage and highlight its potential as both a diagnostic biomarker and therapeutic target for OSA-related cardiovascular complications [129].

Thus, in both OSA and COPD, hypoxia, oxidative stress, and inflammation form a tightly interconnected network that mutually amplifies each component. Continuous positive airway pressure (CPAP) therapy, the gold standard for OSA treatment, effectively minimizes the frequency and severity of hypoxic episodes and reduces apneas and hypopneas while lowering oxidative stress [130,131]. However, the damage caused by oxidative stress in OSA patients is not limited to the airway; it extends to vital organs, including the cardiovascular system. Chronic oxidative damage is a key factor linking OSA to conditions such as hypertension, atherosclerosis, and other cardiovascular diseases [132]. Despite the beneficial effects of CPAP therapy on reducing hypoxic episodes, it does not directly address the underlying oxidative stress or reverse the oxidative damage already incurred. Indeed, some individuals may continue to exhibit residual oxidative stress despite consistent and effective use of CPAP [133].

### Do Antioxidant Therapies Effectively Prevent Symptoms of COPD and OSA?

Despite the well-established role of oxidative stress in COPD and OSA, antioxidant therapies have shown limited clinical success to date. N-acetylcysteine (NAC), a glutathione precursor and mucolytic agent, has demonstrated beneficial effects using in vitro and in vivo COPD models [134]. However, these effects have not been clearly confirmed in clinical studies. Indeed, it has been shown that long-term treatment with high-dose NAC neither significantly reduced the annual rate of total exacerbations nor improved lung function in patients with mild-to-moderate COPD [135]. Other antioxidant compounds, such as vitamin C, have also been reported to increase the levels of antioxidant enzymes in serum and improve lung function [136]. Similarly, other articles suggest that supplementation with vitamins and antioxidants may have a beneficial effect on COPD symptoms, respiratory function, exacerbation, and quality of life [137]. Nevertheless, there is not enough clinical evidence to recommend antioxidant vitamins as standard therapy for COPD, mainly due to the lack of robust and large-scale randomized controlled trials demonstrating long-term efficacy and safety. Overall, the limited clinical success of antioxidant therapy underscores the complexity of redox regulation in COPD and highlights the need for more targeted and disease-specific antioxidant strategies. In some COPD patients, particularly those with overlap syndrome [138] or acute hypercapnic respiratory failure [139], CPAP has demonstrated clinical benefits. In such cases, CPAP may improve gas exchange, enhance ventilatory efficiency, and mitigate nocturnal hypoxemia, which is a key contributor to oxidative stress.

In line with this, the combination of polyphenol-rich antioxidants sourced from *Aronia melanocarpa* berries with CPAP therapy has shown synergistic benefits in the treatment of OSA by lowering the levels of proinflammatory cytokines, such as TNF-α and IL-6, which are commonly elevated in OSA patients [140]. This anti-inflammatory effect may contribute to improved cardiovascular health and a lower risk of long-term complications. Moreover, the polyphenols in *Aronia* enhance endothelial function by increasing nitric oxide (NO) availability. This supports vascular tone regulation and helps reduce blood pressure, thereby potentially lowering the risk of atherosclerosis and other cardiovascular issues, supporting improved overall health outcomes in OSA patients [133]. Similar studies show that tempol, a membrane-permeable radical scavenger, known to be a stable nitroxide, effectively reduces inflammation and oxidative stress, significantly alleviating IH-induced lung injury by modulating the miRNA-145-5p/Nrf2 signaling pathway [141]. Additionally, experimental studies in animal models have shown that antioxidant vitamins may help lower oxidative and carbonyl stress, key contributors to cardiovascular complications in OSA, although these studies did not assess cardiovascular remodeling [131]. Thus, the combination of CPAP with antioxidant therapies might be a potential therapeutic approach to reduce the risk of health complications by further reducing oxidative stress in OSA.

## 4. Oxidative Stress and Inflammation in Comorbidities of Hypoxemic Respiratory Diseases (COPD and OSA)

### 4.1. Role of Oxidative Stress and Inflammation in Cancer Associated with Respiratory Diseases

A growing body of evidence highlights the pivotal role of inflammation in cancer development, particularly by facilitating key processes involved in tumor progression [142]. Inflammation contributes to tumor initiation and promotes the accumulation of oncogenic mutations. Furthermore, inflammatory conditions elevate oxidative stress, resulting in enhanced DNA damage and increased mutation rates [143]. Inflammatory mediators are also implicated in processes such as epithelial to mesenchymal transition (EMT) and epigenetic alterations that favor oncogenesis. In addition, chronic inflammation leads to immune exhaustion, fostering the development of an immunosuppressive tumor microenvironment and facilitating immune evasion [144]. Patients with CRDs, including COPD and OSA, typically present a persistent low-grade inflammatory state, partly driven by hypoxemia [38,120,145].

In the context of COPD, the combination of chronic hypoxia, oxidative stress, and inflammation promotes a tumor-promoting environment in the airways, especially increasing the risk of lung cancer [146]. Although lung cancer can occur regardless of airflow limitation severity, numerous studies have underscored chronic inflammation as a central factor in promoting cellular transformation and disrupting immune regulation, thereby facilitating tumor initiation and progression [120,147]. In COPD patients, inflammatory processes are thought to support carcinogenesis via several mechanisms, including oxidative damage, immune dysregulation, genomic instability, and tissue remodeling [148,149]. In particular, sustained production of ROS, driven by cigarette smoke and repeated hypoxia–reoxygenation, contributes to oxidative DNA damage, lipid peroxidation, and protein carbonylation, all of which impair cellular homeostasis and promote genomic instability [150]. Moreover, chronic inflammation fosters immune dysregulation, which includes impaired immune surveillance and persistent release of proinflammatory cytokines such as TNF-α, IL-6, and IL-1β [151]. These cytokines activate key transcription factors like NF-κB and STAT3, which promote cell proliferation, angiogenesis, and resistance to apoptosis, contributing to malignant transformation [151]. Additionally, repeated cycles of epithelial injury and repair, driven by both oxidative stress, can lead to tissue remodeling and EMT processes known to support tumor invasion and metastasis [152].

Similarly, evidence indicates that OSA is associated with increased cancer incidence, aggressiveness, and mortality [32]. IH experienced during sleep in OSA patients induces oxidative stress and promotes tumorigenic processes, including angiogenesis and epithelial-to-mesenchymal transition [35,153,154,155]. More importantly, there is solid evidence elucidating how the immune surveillance in OSA patients is disrupted due to chronic inflammation, which modulates immune cell subsets and their immune functionality. For instance, a switch to tumor-associated macrophages and a defect in natural killer (NK) cell maturation have been reported as well as suppression of T cell function mediated by overexpression of immune checkpoint axes [35,156,157,158,159].

### 4.2. Role of Oxidative Stress and Inflammation in Cardiovascular Complications Associated with CRDs

Chronic inflammation is a central pathological mechanism implicated in a wide range of metabolic and cardiovascular disorders. Defined as a prolonged and persistent immune activation, chronic inflammation results in tissue injury, oxidative stress, mitochondrial dysfunction, and disruption of physiological homeostasis [160,161,162]. Several inflammatory pathways link respiratory diseases with cardiometabolic comorbidities, with particular emphasis on NF-κB signaling, NLRP3 inflammasome activation, and oxidative stress [162,163]. Although both hypoxemia and ROS are known to stimulate NF-κB and NLRP3, current evidence points to NLRP3 as a major driver of systemic inflammation [164,165]. Sterile stimulation activates the inflammasome complex, enhancing the production of inflammatory cytokines such as IL-18 and IL-1β [166]. These cytokines further exacerbate oxidative stress and hypoxemia. Altogether, chronic inflammation contributes to every phase of cardiovascular disease development, from early onset to overt clinical complications [167,168]. Furthermore, in CRDs, inflammation acts as a persistent noxious stimulus that promotes tissue remodeling, exacerbates inflammatory responses, and induces immune dysregulation. COPD is characterized by persistent inflammation of the airways and lung parenchyma, which intensifies during acute exacerbations and is also linked to systemic inflammation. This inflammation occurs in response to inhaled irritant particles, such as cigarette smoke, which tend to deposit in the lungs and may activate surface macrophages and airway epithelial cells. These cells, in turn, release various chemotactic mediators that attract circulating neutrophils, monocytes, and lymphocytes into the lungs. Indeed, inflammation in COPD is marked by an increased presence of alveolar macrophages, neutrophils, and T-lymphocytes recruited from the bloodstream [169,170,171]. As far as is known, cardiovascular disease and COPD share various risk factors, including smoking, and overlapping mechanisms such as accelerated aging, making cardiovascular mortality more common than respiratory failure in COPD patients [172,173]. Many inflammatory mediators have been implicated in COPD as well as in cardiovascular problems, including lipids, free radicals, cytokines, chemokines, growth factors, and air pollution, and all these are able to activate NF-κB and the NLRP3 inflammasome, leading to an inflammatory response. Indeed, the incidence of major adverse cardiovascular events is 25% higher in patients with COPD compared to those without the condition [174]. Although shared risk factors such as age, smoking, and socioeconomic status contribute to this association, they do not fully explain it. Pathophysiological mechanisms such as hypoxemia, oxidative stress, and systemic inflammation are likely key contributors. In line with this, a systematic review by Chen et al. reported that individuals with COPD are at significantly higher risk of developing cardiovascular disease than the general population (OR 2.46, 95% CI 2.02–3.00) [100,175,176].

OSA patients also exhibit a chronic inflammatory phenotype characterized by oxidative stress and elevated levels of proinflammatory cytokines and adhesion molecules [177]. This inflammatory state is known to induce or exacerbate cardiovascular and metabolic comorbidities such as type 2 diabetes mellitus, dyslipidemia, atherosclerosis, and ischemic heart disease [18,178,179,180]. This “sterile” inflammation occurs in response to damage-associated molecular patterns (DAMPs), such as high-mobility group box 1 protein (HMGB1) and oxidized low-density lipoprotein (oxLDL) [181], often as a consequence of IH and oxidative stress [182,183]. These stimuli activate inflammatory pathways, including NF-κB and the NLRP3 inflammasome [128,184]. The inflammasome activation results in increased levels of the inflammatory cytokines IL-1β and IL-18 in combination with elevated release of the major trigger of coagulation cascade, tissue factor, enhancing systemic inflammation and coagulability [185,186]. Additionally, oxLDL potentiates NF-κB signaling and promotes foam cell formation, contributing to the progression of atherosclerosis [187]. Altogether, this network of IH, oxidative stress, and systemic inflammation fosters endothelial dysfunction and promotes cardiovascular complications in OSA patients.

### 4.3. Role of Oxidative Stress and Inflammation in Pulmonary Hypertension Associated with CRDs

Pulmonary hypertension (PH) is one of the most significant cardiovascular complications associated with CRDs. This condition is defined by an elevation in mean pulmonary arterial pressure exceeding 20 mmHg [188]. Group 3 PH, as classified by the World Symposium on Pulmonary Hypertension, encompasses PH related to CRDs and represents one of the most prevalent subtypes. It includes conditions with diverse etiologies such as COPD, interstitial lung diseases (e.g., pulmonary fibrosis), non-parenchymal restrictive disorders (e.g., OSA), hypoxia without underlying lung disease (e.g., high-altitude exposure), and developmental lung conditions (e.g., bronchopulmonary dysplasia) [189].

The development of PH in the context of CRDs is associated with increased morbidity, reduced quality of life, and poorer prognosis [190]. The underlying pathophysiology is complex and multifactorial, involving disease-specific mechanisms [191]. Hypoxia represents a major common factor in these conditions and much of the insight concerning the molecular mechanisms responsible for pulmonary vasculopathy in CRDs comes from experimental animal models with exposure to hypoxia [192]. Acute hypoxia induces a compensatory response known as hypoxic pulmonary vasoconstriction (HPV), aimed at optimizing ventilation–perfusion matching. However, sustained hypoxia promotes persistent HPV and vascular remodeling, resulting in elevated pulmonary vascular resistance and PH development [192,193]. The involvement of ROS in HPV has been widely documented, although some controversy remains regarding whether ROS levels increase or decrease under hypoxic conditions. Current evidence predominantly supports an increase in ROS, which activates multiple downstream effectors that elevate intracellular calcium and induce contraction of PASMCs [194,195,196,197]. In chronic hypoxia, persistently elevated ROS levels are well-established contributors to pulmonary arterial remodeling and enhanced pulmonary vasoconstriction, as observed in chronic-hypoxia-induced PH [198,199,200]. Similar mechanisms have also been identified in chronic IH, a widely used model for OSA-associated PH [201,202].

Mitochondria-derived ROS are considered pivotal in HPV [196,197,203], although other sources, such as NOX, may amplify the response [194,195]. Mitochondrial respiratory chain [204] and members of the NOX family [205], particularly the isoforms NOX2 and NOX4, are considered key sources of this hypoxic ROS generation. NOX2 is primarily expressed in phagocytes and has been associated with inflammatory responses [206], while NOX4 is constitutively active in vascular endothelial and smooth muscle cells and is implicated in vascular remodeling under oxidative stress conditions [207,208]. Intriguingly, there is also evidence of decreased ROS in PASMCs after chronic hypoxia [197]. Excessive oxidative stress has also been postulated as a key driver of lung fibrosis [209,210]. Indeed, TGF-β, a key mediator of pulmonary fibrosis and associated pulmonary vasculopathy, enhances the production of ROS through mitochondrial pathways and NOX activation [209]. Conversely, ROS can further stimulate the release of TGF-β from alveolar cells, creating a self-amplifying cycle [211]. Moreover, the protective effects of phosphodiesterase type 5A (PDE5A) inhibition in bleomycin-induced pulmonary fibrosis and PH were associated with inhibition of ROS formation [212]. In the perinatal period, ROS-mediated injury to the developing lung is also recognized as a critical factor in the pathogenesis of neonatal pulmonary vascular diseases, including persistent pulmonary hypertension of the newborn and bronchopulmonary dysplasia [195,213,214,215].

As already mentioned, inflammation is increasingly recognized as a key contributor to pulmonary vascular disease and plays a significant role in the pathogenesis of various forms of PH, including those associated with CRDs [191,193,216]. Many immune cells, and multiple cytokines, adhesion molecules, and differentiation factors, have been suggested to participate in the complex inflammatory responses promoting the pulmonary vascular remodeling characteristic of PH [191,193,217]. Thus, elevated levels of inflammatory cytokines and chemokines, including TNF-α, IL-1β, IL-6, Il-8, IL-17, IL-21, CCl2, and CX3CL1, among others, originating from a variety of inflammatory cell types are considered hallmarks of CRD-associated PH [201,218,219,220,221,222]. Extensive evidence highlights monocytes and macrophages as central mediators of lung inflammation and vascular remodeling in hypoxia-induced PH [221,223,224,225]. Upon hypoxic exposure, circulatory monocytes are recruited into the lungs where they differentiate into interstitial macrophages. Both non-classical [221,223] and classical monocytes [222,226] have been proposed as precursors of recruited interstitial macrophages. The expression of TSP-1 in these recruited interstitial macrophages was shown to activate TGF-β, resulting in enhanced pulmonary vasoconstriction and remodeling [222,224,226]. There is also the involvement of lymphoid cells in hypoxia-induced PH. Thus, CD4^+^ T cells, specifically Th17 cells, have been demonstrated to contribute to the development of hypoxia-induced PH in murine models [220,227] and their inhibition lowers right ventricle systolic pressure in established PH [227]. Similarly, Th17 cells have been suggested to play a detrimental role in PH secondary to lung fibrosis [201].

In addition to hypoxia, other pathogenic environmental factors involved in the development of respiratory diseases such as cigarette smoke, hyperoxia, and others (air pollution, chemicals, and toxins such as asbestos or heavy metals) may produce pulmonary vascular injury through increased ROS or inflammation. Thus, recent evidence suggests that cigarette smoke, a major risk factor in COPD, may directly impact the pulmonary vasculature, independent of low oxygen levels [228]. This is particularly relevant in early-stage COPD and even in smokers without airway obstruction. In advanced COPD, the mean pulmonary artery pressure shows only a weak correlation with hypoxemia and no correlation with airflow limitation [229]. Additionally, vascular changes such as medial thickening are seen in mild COPD and in smokers without airway obstruction [230]. Furthermore, animal studies show that cigarette-smoke-induced PH can occur before emphysema development and without hypoxia [231]. These findings point to early pulmonary artery involvement, likely driven by cigarette smoke itself, even before hypoxia plays a role. This is critical, as CS can compromise epithelial barriers, allowing toxic substances to reach and damage nearby blood vessels [232]. Cigarette smoke also induces pulmonary vascular dysfunction through the secretion of inflammatory molecules and increased ROS production [228]. In particular, cigarette smoke promotes the production of mitochondria-derived ROS, affecting the redox state of the soluble guanylyl cyclase, which severely impairs the NO-soluble guanylyl cyclase signaling pathway in the pulmonary vasculature [8]. Using a murine model of nicotine/hypoxia coexposure to better illustrate the pathological conditions of COPD-PH, Truong et al. have recently identified a major role of Rieske iron–sulfur protein (RISP)-mediated mitochondrial ROS and NF-κB-dependent inflammation in PH development [233]. Other sources of ROS such as xanthine oxidoreductase have also been shown to contribute to the harmful effects of cigarette smoke in pulmonary vascular endothelial cells [234]. In addition, cigarette smoke has been shown to promote dysregulated inflammatory signaling due to increased recruitment of inflammatory cells or ROS-induced inflammation in pulmonary arteries [228,233,235]. An excess of ROS production is also recognized as a critical factor for hyperoxia-induced pulmonary vascular damage in several conditions such as respiratory distress syndrome [236] or pulmonary bronchodysplasia [237].

Many efforts have been made to target oxidative stress and the inflammatory component underlying the pulmonary vascular injury linked to CRDs (Table 1). Thus, targeting ROS with unspecific antioxidants such as NAC [238] or with strategies to specifically reduce mitochondrial ROS [198,199,204] show protective effects in chronic hypoxia-PH. Similarly, antioxidants such as the glutathione peroxidase mimetic ebselen [239] or the NOX inhibitor apocynin [240] show protective effects on vascular dysfunction due to cigarette smoke exposure. Other strategies such as dietary antioxidants, Nrf2 activators, or nitrative stress inhibitors have shown benefit in animal models of COPD [241] and might be useful in ameliorating the associated pulmonary vascular dysfunction. Alternatively, numerous intervention strategies for inflammation have been proven as effective in attenuating hypoxic PH. These include, among others: glucocorticoids [222], blocking IL-6 [218,219,220], NF-κB activation [204], CX3CR1 [221], the SDF-1/CXCR4 axis [242], the CCR2-CCL2 axis [222], inhibiting macrophages [220], switching macrophage polarity toward an anti-inflammatory phenotype [225], or adoptive transfer of M2 regulatory macrophages [243].

Despite substantial evidence supporting the pathogenic role of oxidative stress and inflammation in pulmonary-vascular-damage-associated CRDs, and the demonstrated benefits of therapies targeting these pathways in preclinical studies, neither antioxidants nor anti-inflammatory drugs are indicated in PH associated with CRD. So far, clinical trials with antioxidants in COPD have shown disappointing results due to the lack of proven efficacy or occurrence of adverse effects [241]. The development of more effective antioxidants or anti-inflammatory drugs with limited side effects confirmed in clinical trials will determine whether these strategies can be successfully implemented in clinical practice.

## 5. Relevance of AI in Respiratory Medicine

Machine learning (ML) is a subfield of artificial intelligence that enables computer systems to learn patterns from data and make predictions or decisions without being explicitly programmed. At the core of any ML system is a model, typically represented by a function **h**, which maps an input **x** (e.g., patient data, medical images, or sensor readings) to an output (e.g., a diagnosis, risk score, or recommended treatment). This function **h** is not predefined; rather, it is learned from a dataset during a training phase, where the model adjusts its internal parameters to approximate the underlying relationship between inputs and **outputs**.

In medicine, the application of ML has grown exponentially, evolving from traditional methods to advanced deep learning (DL) models. Traditional ML models learn patterns from clinical features such as symptoms, spirometry values, or imaging metrics, demonstrating effectiveness in both diagnostic and prognostic predictions for conditions such as COPD and OSA, and more modern DL models can learn from images, audio, or complex tabular data [41,42,247,248]. Also, so-called foundational models have been important to empower AI applications in many fields of medicine. Foundational models are mainly based on the Transformer architecture [249] that are trained with huge amounts of data for general purpose tasks and that can be refined or transferred to specific tasks with a more reasonable amount of data and computing resources. This architecture allows the training of large AI systems integrating data from multiple sources (tabular, text, image, and audio) enhancing the human–machine interaction with specialized chatbots [250,251], generative models capable of synthesizing data with to train and evaluate AI systems [252,253], and a huge potential for analysis and exploration of molecular pathways in order to improve diagnosis and prognosis and also to find comorbidities [254,255,256]. Moreover, the role of AI in personalized and precision medicine is paramount, and its value has been increasing in recent times [257,258,259].

The application of AI in respiratory medicine faces several challenges in the coming years. Table 2 outlines some of these key challenges and highlights their importance for advancing the field.

### 5.1. Databases for Respiratory AI Research

ML applications in COPD and OSA research have been made possible by recent publications of extensive clinical datasets. For COPD, some of the most prominent cohorts include COPDGene [267], ECLIPSE [268], SPIROMICS [269], and CanCOLD [270], which offer clinical data, genetic profiles, spirometry results, biomarkers, and chest computed tomography (CT) imaging from patients with and without COPD, covering diverse stages of disease severity. Additionally, intensive care databases like MIMIC-III [260], MIMIC-IV [261], eICU [262], and HiRID [263] provide detailed respiratory data specific to patients with critical respiratory conditions, including those with acute exacerbations of COPD [271]. In sleep apnea research, prominent databases, including Apnea-ECG [272], UCD Sleep Apnea Database [273], and SHHS [274], offer detailed polysomnographic recordings, ECG, EEG, EOG signals, apnea event annotations, and extensive longitudinal clinical data, enabling a wide range of analytical approaches.

### 5.2. Predictive Machine Learning for Diagnosis and Prognosis

Regarding traditional AI methods, temporal physiological signals from portable sensors have proven effective in diagnosing and predicting outcomes in respiratory diseases. In COPD, models like XGBoost, RF, and SVM have achieved strong performance using data from capnography, air quality monitors, remote spirometry, and electronic stethoscopes [275,276,277]. For OSA, inputs from polysomnography (e.g., ECG, airflow, muscle activity), as well as voice and tracheal sounds [42,248], have been effectively analyzed using SVM, RF, and LR models [219,220,221]. Radiomics features from chest CT scans have shown strong diagnostic and prognostic value in COPD and OSA, particularly when analyzed with ML models like LR, SVM, and RF, outperforming demographic models and basic emphysema measures [278,279,280]. Radiomics has also helped distinguish COPD from asthma and predict disease progression, especially when combined with clinical data [281,282], sometimes surpassing CNN-based methods [283]. Tabular clinical data remains widely used with ML models such as XGBoost, RF, and LightGBM to predict outcomes like respiratory failure, ventilation need, mortality, and exacerbations in COPD, often integrating demographics, comorbidities, and test results [284,285,286]. In OSA, clinical and anthropometric features have enabled accurate prediction of the apnea–hypopnea index and phenotype classification using neural networks, SVMs, RF, and LR models [42,248,287]. In parallel, there is growing interest in enhancing these models by incorporating molecular biomarkers to enrich predictive capabilities. In recent years, various studies have highlighted the role of molecular factors such as *HIF1A* (the gene encoding HIF-1α), markers of chronic oxidative stress (e.g., TXN, EGR1, CDKN1A), receptors associated with persistent inflammation such as TLR2, and ADM, particularly its mid-regional form, in the pathophysiology of both COPD and OSA. Their relevance in pulmonary diseases has been validated using classical statistical analyses (ROC curves, regression, etc.) as well as ML algorithms, demonstrating their potential as biomarkers for AI-based models [288,289,290,291,292,293]. Currently, emerging studies are exploring the integration of these features into ML and DL models to enhance risk stratification and support clinical decision making [294,295]. Nevertheless, significant limitations persist due to the scarcity of standardized molecular data across cohorts. Recent DL advances have enabled models like CNNs, RNNs, and transformers to achieve expert-level performance in clinical tasks [296,297,298,299,300,301], with some gaining regulatory approval [296]. In COPD and OSA, DL models using temporal signals (e.g., lung sounds, ECG, PSG) have shown high accuracy for classification and event detection [302,303,304,305,306,307,308,309,310,311,312,313], while CNNs and GCNs applied to chest CT and X-rays have outperformed traditional ML in diagnosis, severity staging, and mortality prediction [279,297,314,315,316,317,318,319,320,321]. For OSA, CNN-based methods have accurately detected apnea using various image-based signal representations [307,308,310,311,312,322,323]. Although DL has also been used with clinical tabular data via MLPs and attention-based models like HiTANet [324,325], traditional ML remains competitive, especially in low-dimensional, standardized datasets [42,247,248].

### 5.3. Foundational Models for Diagnosis and Prognosis

Foundational models are large, Transformer-based neural networks trained with self-supervised learning on massive datasets, enabling adaptation to downstream tasks through fine-tuning [298]. Unlike specialized models tailored to specific tasks, foundational or generalist models are designed to handle a wide range of inputs and tasks, representing a shift toward more versatile AI in medicine. Notable examples include ChatGPT 3.5 (used in clinical text generation and exams), CLIP [326], and BioBERT [327] for biomedical text mining. In respiratory medicine, foundational models offer a promising path for integrating multimodal data such as text, images, and biosignals in unified diagnostic frameworks.

In audio analysis, the OPERA framework [328] introduced three models—OPERA-CT, OPERA-CE, and OPERA-GT—trained on over 136,000 audio samples and benchmarked across 19 respiratory health tasks (e.g., COPD detection, pulmonary function estimation), performing competitively against baselines like OpenSMILE [329], VGGish [330], AudioMAE [331], and CLAP [332]. In medical imaging, M3FM [333] is a general-purpose model trained on over 128,000 3D CT scans and multimodal data using a question–answering framework. It significantly outperformed prior models such as Sybil [334], Tri2D-Net [335], and GPT-4o, especially in tasks like emphysema and lung cancer detection. For clinical text and EHR analysis, BEHRT [250] was trained on data from ~1.6 million patients in the UK CPRD database [336]. It predicted the onset of 301 conditions (including COPD), outperforming previous models such as Deepr [337] and RETAIN (RNN with reverse-time attention) [338], particularly in first-incidence disease prediction.

Foundational AI models, particularly those based on Transformer architectures, have demonstrated transformative potential in the integration and interpretation of high-dimensional molecular data for the diagnosis and prognosis of complex diseases, including respiratory disorders. These models are uniquely suited to handling multiomics inputs—such as gene expression, proteomics, and epigenetic profiles—alongside clinical and imaging data, enabling the discovery of novel molecular patterns associated with disease phenotypes and progression [254,255,256]. In respiratory diseases such as COPD and obstructive sleep apnea (OSA), foundational models facilitate the identification of shared molecular pathways and stratify patients according to their genetic and inflammatory signatures, supporting early diagnosis and risk prediction. By leveraging self-supervised learning and transfer learning, these models can be adapted across datasets and conditions, improving generalizability in heterogeneous patient populations, including the exploration of comorbidities with other diseases. Furthermore, foundational models enhance the potential for precision and personalized medicine by predicting individual responses to therapy based on molecular profiles, thereby optimizing treatment decisions and improving outcomes in chronic respiratory conditions [41].

Together, these models demonstrate strong potential for unifying multimodal AI tools in chronic respiratory care, offering adaptability and improved performance across diverse tasks and data types.

### 5.4. Ethical, Legal, and Social Aspects of AI Respiratory Medicine

AI, particularly generative AI, is becoming an increasingly powerful tool in medicine, with the potential to function as a comprehensive assistant or even as an autonomous system in clinical environments. These models can handle multiple tasks with minimal adjustment, suggesting a future in which medical AI may play an integral role in therapeutic decision making. However, despite this promise, AI also presents considerable risks and challenges, especially when used in high-stakes contexts such as healthcare. The lack of explainability and biased results of AI-based systems are important concerns for both research community and society. Many AI models, and particularly generative AI models, are trained using mathematical optimization techniques, which can sometimes make the interpretation of their results challenging. AI models learn from data, which may underrepresent specific populations, leading to less accurate predictions for specific demographic groups [339]. This could perpetuate or even exacerbate existing disparities in healthcare delivery and outcomes. Regulators and practitioners increasingly demand interpretability or, at minimum, reliable confidence metrics before integrating AI into clinical workflows [298,340].

Beyond these, there are growing concerns about privacy, security, and accountability. AI systems trained on patient data must comply with strict data protection laws such as HIPAA and GDPR. They must also be resilient against adversarial attacks and ensure robust mechanisms for ethical and legal responsibility when outcomes are incorrect or harmful. To mitigate these risks, regulatory frameworks are evolving. The European Union has classified these systems as high-risk technologies, subject to strict compliance requirements under the EU Artificial Intelligence Act [341]. This Act, the world’s first comprehensive AI legislation, adopts a risk-based approach and mandates the following for medical AI applications (e.g., diagnostic tools for COPD or OSA): (1) adequate risk assessment and mitigation plans. (2) High-quality training datasets to minimize bias. (3) Logging capabilities to ensure traceability. (4) Comprehensive documentation for regulatory review. (5) Clear deployment instructions and information. (6) Human oversight mechanisms. (7) High levels of robustness, accuracy, and cybersecurity. In contrast, the United States currently lacks a comprehensive AI law. However, the FDA leads the regulation of AI- and ML-based medical devices. The FDA requires clinical evidence of safety and efficacy, typically through validation studies, before approving or authorizing such systems. Notably, the FDA has approved autonomous AI systems [296], marking an important precedent.

### 5.5. Integration of the AI in Practitioner’s Decisions

AI can significantly enhance clinical decision making but must remain a support tool, with healthcare professionals retaining ultimate responsibility. Decision theory provides a formal framework for managing uncertainty in high-stakes fields like oncology and respiratory diseases such as COPD and OSA [342]. For AI to aid practitioners effectively, its outputs must be interpretable—something enabled by probabilistic models that offer well-calibrated predictions and support personalized, cost-effective care [343]. However, the utility of these models depends on their calibration, as misaligned probabilities can lead to harmful over- or underestimations that may affect treatment, diagnosis, or prognosis [344,345]. Techniques like Platt scaling, isotonic regression, or Bayesian postprocessing are thus vital for ensuring reliable predictions, especially in imbalanced clinical data [346]. Integrating calibrated models into decision-theoretic frameworks enhances interpretability, trust, and actionable decision making in healthcare and offers practitioners an invaluable decision support tool.

## 6. Conclusions, Future Research, and Directions

COPD and OSA are both hypoxemic lung diseases that share overlapping pathophysiological mechanisms, including hypoxia-induced oxidative stress and systemic inflammation, which exacerbate disease progression and comorbidities. In OSA, recurrent apneic episodes during sleep result in IH, which in turn promotes excessive ROS production and systemic inflammation. Similarly, in COPD, chronic hypoxia and exposure to environmental insults such as cigarette smoke promote oxidative stress and a persistent inflammatory response. These common pathways contribute to endothelial dysfunction, vascular remodeling, and elevated cardiovascular risk. While antioxidant therapies offer promising strategies for mitigating oxidative-stress-related lung damage, a thorough understanding of the evolving role of ROS in pulmonary inflammation is essential for the development of more effective treatments. In addition, the high prevalence of pulmonary hypertension in both conditions underscores the need to elucidate shared mechanisms and identify new therapeutic strategies.

In this respect, understanding the role of HIFs in CRDs provides opportunities for targeted therapies. While initially adaptive, sustained HIF activation may exacerbate inflammation and tissue remodeling, suggesting a dual role that must be carefully navigated in therapeutic design. Despite the promise of antioxidant and anti-inflammatory treatments, their clinical translation remains limited due to the complexity of redox and inflammatory signaling networks, highlighting the need for integrative and multidisciplinary approaches.

In this context, AI has emerged as a powerful tool to manage the clinical and biological complexity of OSA and COPD. ML and DL models enable accurate phenotyping, risk stratification, and treatment personalization by integrating multimodal data, including clinical, imaging, and physiological signals. AI-based models have improved the classification of disease subtypes, predicted treatment responses, and consistency in diagnosis, particularly in pulmonary function testing and imaging analysis. Foundational models like OPERA and M3FM exemplify AI’s potential to unify heterogeneous data sources and bridge the gap between molecular insights and clinical decision making, including comorbidities with other diseases, and also the use of all the gathered knowledge in personalized medicine. Despite rapid progress, the integration of AI into routine clinical practice faces critical challenges, including limited availability of high-quality data, suboptimal model accuracy for diagnosis and prognosis, regulatory hurdles, and the complex task of embedding AI decision making into existing clinical workflows. In conclusion, the convergence of OSA and COPD pathophysiology reflects shared systemic consequences of hypoxia, oxidative stress, and inflammation. Advances in molecular understanding, particularly of HIF signaling, and the integration of AI into clinical workflows offer promising avenues for precision medicine. Interdisciplinary strategies combining biomedical research and computational innovation are essential to improve outcomes in these prevalent and complex respiratory diseases.

## Figures and Tables

**Table 1:** Effects of treatment with antioxidants or anti-inflammatory agents on pulmonary vascular damage associated with CRD in preclinical studies.

Drug	Target	Experimental Model	Main Findings	Reference
N-acetylcysteine	Thiol antioxidant	Chronic hypoxic rats	Reduced risk of hypoxic PH development	[238]
N-acetylcysteine	Thiol antioxidant	Cigarette smoke (CS)-exposed mice	Partial prevention of impaired pulmonary vascular disfunction	[228]
Apocynin	Non-selective NOX inhibitor	CS-exposed mice	Prevention of CS-induced endothelial dysfunction	[110]
MitoTEMPO	Mitochondrial antioxidant	CS-exposed pulmonary arteries	Prevention of impaired nitric-oxide-mediated pulmonary vasodilation	[8]
MitoTEMPO	Mitochondrial antioxidant	Human pulmonary arterial endothelial cells	Prevention of hypoxic-induced mitochondrial ROS production	[198]
Ebselen	Glutathione peroxidase mimetic	CS-exposed mice	Prevention of endothelial dysfunction and CS-induced lung inflammation	[239]
Allopurinol	Xanthine oxidase inhibitor	Chronic hypoxic neonatal rats	Attenuated hypoxia-induced pulmonary vascular remodeling	[244]
Tetracaine	Ryanodine receptor antagonist	Chronic hypoxic mice	Prevention of hypoxic PH development	[204]
S107	Stabilizer of ryanodine receptor 2/FKBP12.6 complex	Chronic hypoxic mice	Prevention of hypoxic PH development	[204]
Pyrrolidine dithiocarbamate	Inhibition of NF-κB activation	Chronic hypoxic mice	Prevention of hypoxic PH development	[204]
Tracolimus (FK506)	Inhibits calcineurin, blocking the activation of NFAT	Chronic hypoxic mice	Low dose protects while high dose facilitates PH development	[204]
MR16-1	Monoclonal anti-IL-6 receptor antibody	Chronic hypoxic mice	Amelioration of hypoxic PH development	[220]
AMD3100	CXCR4 antagonist	Chronic hypoxic mice	Prevention and reversion of neonatal hypoxic PH development	[242]
Antistromal derived factor-1 (SDF-1) antibody	Blockade of SDF-1	Chronic hypoxic mice	Attenuation of neonatal hypoxic PH development	[242]
Dexamethasone	Glucocorticoid receptor	Human individuals exposed to high altitude	Dexamenthasone prophylaxis may mildly mitigate acute high-altitude PH	[222]
Fucoxanthin	Natural antioxidant	Intermittent chronic hypoxic rats	Attenuation of intermittent hypoxic-induced PH	[245]
Grape seed procyanidin extract	Natural antioxidant	Intermittent chronic hypoxic rats	Attenuation of intermittent hypoxic-induced PH	[246]

Abbreviations: CS: Cigarette smoke; PH: Pulmonary hypertension; NOX: NADPH oxidase; SDF-1: Stromal derived factor-1.

**Table 2:** Main challenges of AI systems applied to respiratory medicine.

Scarcity of public databases	Public databases are key for AI training and evaluation and foundational models. MIMIC-III [260], MIMIC-IV [261], eICU [262], and HiRID [263] provide detailed respiratory data. Still, large-scale multimodal datasets with respiratory features are needed.
Performance/Accuracy for diagnosis and prognosis	Accurate systems are crucial for trustworthy AI in medical decisions. COPD and OSA models still show detection accuracies below 80% [41,42,247].
Ethical, legal, and social aspects (ELSAs)	AI must be human-centric, considering ethical, legal, and social impacts from the start. The EU AI Act highlights early integration of ethics, regulation, and societal impact.
Lack of international standards	International coordination is key for shared protocols and best practices. Standards like ISO/IEC 27001 [264] and ISO/TS 82304-2 [265] guide secure, reliable health data use and trustworthy AI. Common frameworks support multidisciplinary efforts.
Integration in practitioner decisions	AI should support, not replace, practitioner decision making. Bayesian decision theory and calibration have aided high-stakes fields like medicine and forensics [266], being data- and domain-agnostic.
Insufficiency of structured application data	AI in medicine often faces limited real data, i.e., real-world evidence (RWE), due to legal, ethical, and infrastructural barriers. While transfer learning and robust models help, collecting and structuring clinical and molecular RWE remains a challenge.
